# Impact of parathyroidectomy on left ventricular function in end stage renal disease patients

**DOI:** 10.1186/s12882-020-02139-3

**Published:** 2020-11-13

**Authors:** Shaohua Chen, Kaixiang Sheng, Ying Shen, Hua Jiang, Xin Lei, Lihui Qu, Chunping Xu, Jianghua Chen, Ping Zhang

**Affiliations:** 1 Kidney Disease Center, the First Affiliated Hospital, Zhejiang University School of Medicine, Hangzhou, Zhejiang China; 2 Key Laboratory of Kidney Disease Prevention and Control Technology, Zhejiang, Hangzhou China

**Keywords:** ESRD, Left ventricular function, Secondary hyperparathyroidism, Parathyroidectomy

## Abstract

**Background:**

Secondary hyperparathyroidism (SHPT) is a common complication in end-stage renal disease (ESRD) patients, and parathyroidectomy (PTX) is an effective treatment intervention of SHPT. However, the curative impact of PTX on left ventricular function still remains incompletely understood. To evaluate the impact of parathyroidectomy on left ventricular function in ESRD patients, we conducted this retrospective study.

**Methods:**

Between Oct 1, 2010 and Oct 1, 2016, ESRD patients presented with SHPT who underwent parathyroidectomy were enrolled. We retrospectively collected the ultrasonic cardiogram parameter pre- and 1-year post-PTX, and analyzed the influence factor for the overturn of left ventricular hypertrophy (LVH) and the improvement of ejection fraction% (EF%).

**Results:**

In all the patients (135), the main ultrasonic cardiogram parameter dramatically improved after PTX. Compared with pre-PTX, the left ventricular mass (LVM) (172.82 (135.90, 212.91) g vs. 192.76 (157.56, 237.97) g, *p<*0.001) and the left ventricular mass index (LVMI) (107.01 (86.79, 128.42) g/m^2^ vs. 123.54 (105.49, 146.64) g/m^2^, *p<*0.001) significantly declined after 1 year of the PTX. Further, 43.75% patients diagnosed with LVH before the PTX have recovered from LVH. In the subgroup analysis of 35 patients with EF% ≤ 60% pre-PTX, EF% and fractional shortening% (FS%) significantly improved after 1 year of the PTX compared with pre-PTX (EF%: 64.90 ± 7.90% vs. 55.71 ± 4.78%, *p<*0.001; FS% 35.48 ± 6.34% vs. 29.54 ± 2.88%, *p<*0.001), and 82.86% patients underwent an improvement of left ventricular systolic function post 1year of the PTX.

**Conclusions:**

tPTX+AT is an effective curative intervention of secondary hyperparathyroidism and can significantly overturn the LVH and increase the left ventricular systolic function.

## Background

The morbidity of secondary hyperparathyroidism is high in ESRD patients. Among all the complications, cardiovascular events are the leading causes of death, and the mortality caused by cardiovascular disease (CVD) is 10 to 100 times higher than that in the general population [1]. While secondary hyperparathyroidism is an important risk factor of CVD in ESRD, PTX is an effective therapeutic method for severe SHPT unmitigated by medicine therapy. It has been reported that the cardiovascular mortality of patients who underwent successful PTX, registered 37–41% reduction [2, 3]. However, the impact of PTX on left ventricular function remains obscure. Case report and small sample size studies have shown that the EF% and FS% of hemodialysis patients were significantly improved after the PTX [4, 5]. This study was conducted retrospectively to fully explore the impact of PTX on the left ventricular function of dialysis patients.

## Methods

### Patients

The cohort of this retrospective study consisted of ESRD patients with SHPT received PTX from Oct 1, 2010, to Oct 1, 2016, at the First Affiliated Hospital, Zhejiang University School of Medicine. The inclusion criteria of PTX were according to Kidney Disease Improving Global Outcomes (KDIGO) 2009 guidelines [6]. Accordingly, following patients were excluded: those having severe malnutrition, infection or inflammation; severe liver disease and coagulation disorders; complicated with a hematological disease or malignant tumors; having a history of acute cardiovascular and cerebrovascular events within 6 months; the parathyroid hormone (PTH)>100 ng/L within 1 month post- PTX; those who received the kidney transplantion during the follow-up; with follow-up < 1 year; received the PTX because of recurrence and data missing or insufficiency. Patients enrolled were followed for at least 1 year; baseline characteristics including age, sex, dialysis duration, dialysis modality, the primary diseases of ESRD, the number of parathyroid gland removed, the blood pressure pre- and 1-year post-PTX, the cardiovascular drugs used during the follow-up were collected. This study received approval from the Research and Ethics Committee of the First Affiliated Hospital, Zhejiang University School of Medicine.

### Surgical methods and perioperative management

The surgery method employed was total PTX with forearm autotransplantation (tPTX+AT). The parathyroid tissues were forwarded for pathological examination intra- and post-operatively. Tiny pieces of parathyroid tissues were transplanted into patients’ forearms. Post-operatively, all patients were given substantial doses of intravenous calcium, oral calcium, and calcitriol supplement.

### The biochemical parameters

We collected the biochemical parameters including pre-operative serum calcium, phosphate, alkaline phosphatase, parathyroid hormone, and albumin levels; monitored and observed them at 1 week, 1 month, 3 months and 1 year after the PTX.

### The cardiovascular drugs and the blood pressure

We collected the cardiovascular drugs used during the follow-up, including angiotensin-converting enzyme inhibitors (ACEI), angiotensin-receptor blockers (ARBs), calcium blockers, cardiotonic β-blockers, isosorbide mononitrate and trimetazidine during the follow-up, and the blood pressure 1-year post-PTX.

### The ultrasonic cardiogram

We also collected the ultrasonic cardiogram parameter pre- and 1- year post-PTX, including ejection fraction% (EF%), fractional shortening% (FS%), left ventricular end-diastolic diameter (LVDd), interventricular septum thickness (IVST), left ventricular posterior wall end-diastolic thickness (LVPWD), left atrial diameter (LA), aorta (AO), left ventricular end-systolic dimension (LVDs), left ventricular posterior wall end-systolic thickness (LVPWs), ventricular septal end-systolic thickness (IVSs).

We used the Devereux correction formula to calculate the Left ventricular mass index (LVMI).

Left ventricular mass (LVM, g) = 0.8 × 1.04{[LVDd (cm) + IVST (cm) + LVPWd (cm)]^3^-LVDd^3^} + 0.6;

Body surface area (BSA) (m^2^, female) = 0.0073 × height (cm) + 0.0127 × weight (kg) -0.2106;

BSA (m^2^, male) = 0.0057 × height (cm) + 0.0121 × weight (kg) + 0.0882;

LVMI (g/m^2^) = LVM/BSA.

The diagnostic criteria of left ventricular hypertrophy (LVH) are: LVMI≥95 g/m^2^ (female), LVMI≥115 g/m^2^ (male) [7].

The improvement of LV systolic function was defined as the improvement of the EF% measured by echocardiography to an increase of 10% or greater in its absolute value 1-year post-PTX.

### The primary and the secondary evaluation items

The primary evaluation items were: the change of ultrasonic cardiogram parameter pre- and 1-year post-PTX; the influence factor for the overturn of LVH; the influence factor for the improvement of EF%.

The secondary evaluation items included the biochemical parameters change pre- and post-PTX.

### Data analysis

We used the Statistical Package for the Social Sciences (SPSS) version 23.0 (SPSS Inc., Chicago, IL, USA) for statistical analyses. We used Kolmogorov-Smirnov (KS) test to determine the normality of the continuous variables. Continuous variables were presented as mean ± SD or interquartile range, and categorical variables were presented as number and proportion. We used the paired t-test and Wilcoxon signed- rank sum test to check the difference between ultrasonic cardiogram parameters, LVM and LVMI, pre- and at one-year post-PTX. Moreover, We used the multiple logistic regression analysis to determine the influence factor for the overturn of LVH and the improvement of EF% one-year post-PTX. *P* < 0.05 was regarded as statistically significant.

## Results

### Patients’ characteristics

**Fig. 1:**
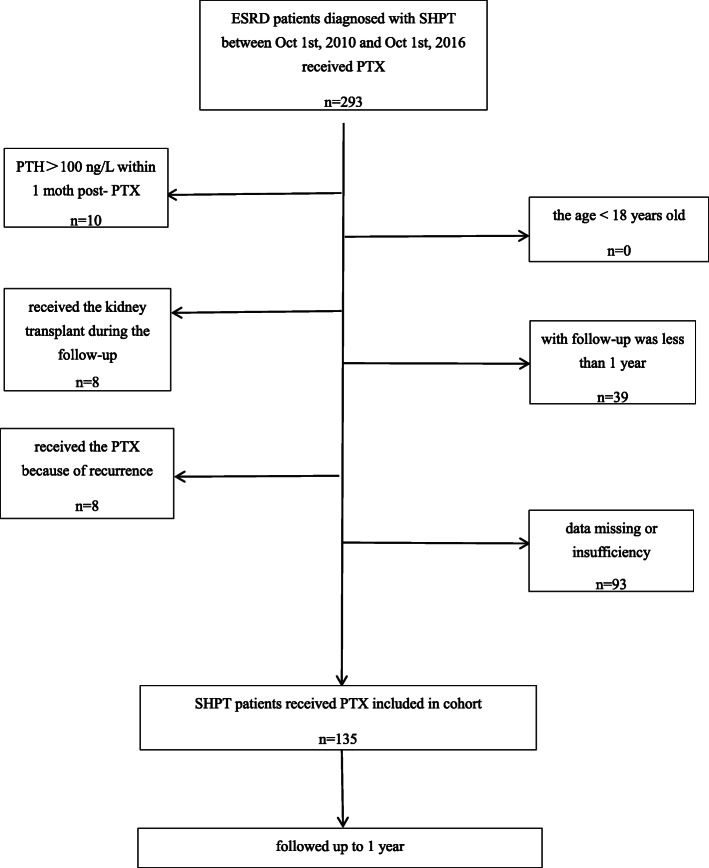
derivation of cohort

There were 293 ESRD patients diagnosed with SHPT between Oct 1, 2010, and Oct 1, 2016 received parathyroidectomy. Out of them, following patients(158) were excluded: 10 patients were excluded because of the PTH>100 ng/L within 1 month post- PTX, 8 because of receiving the kidney transplant during the follow-up, 39 because of the follow-up<1 year, 8 because of receiving the PTX due to recurrence; and 93 because of data missing or insufficiency. In total, 135 ESRD patients (81 male: 54 female) with severe SHPT were included (Fig. 1). The mean age was 45.60 ± 1.0 years, and the media dialysis duration was 6.45(5.0, 9.0) years. There were 121 hemodialysis patients, 12 peritoneal dialysis patients, and 2 ESRD patients who did not start renal replacement therapy when the PTX was performed. The most common primary disease was chronic glomerulonephritis (80%). There were 119 patients (88.1%) had ≥4 parathyroid glands removed, while 16 patients (11.9%) had <4 parathyroid glands removed. Further, all parathyroid glands turned out to be hyperplasia or adenoma by pathological examination. The average systolic pressure pre-PTX was 139.4 ± 22.8 mmHg, while the average diastolic pressure was 88.6 ± 15.4 mmHg; the average systolic pressure 1-year post-PTX was 144.5 ± 23.5 mmHg, and the average diastolic pressure was 80.1 ± 13.3 mmHg. As to the cardiovascular drugs used in the follow-up, 5 (3.7%) patients took ACEI, 30 (22.2%) patients took ARBs, and 50 (37.0%) patients took calcium blockers, only 1 (0.7%) patient took cardiotonics, 48 (35.6%) patients took β-blockers, 11 (8.1%) patients took isosorbide mononitrate, and 8 (5.9%) patients took trimetazidine (Table 1). All the patients were followed up to 1 year.

**Table 1:** demographic and baseline characteristics of the patients

CHARACTERISTIC	NUMBER
Sex	
Male	81(60.0%)
Female	54 (40.0%)
Age	45.60 ± 1.0 year
Dialysis duration	6.45 (5.0,9.0) year
Dialysis modality	
PD	12 (8.9%)
HD	121 (89.6%)
ESRD without dialysis	2 (1.5%)
Primary disease	
CGN	108 (80%)
Polycystic kidney	10 (7.4%)
Hypertensive nephropathy	5 (3.7%)
Diabetic nephropathy	4 (3.0%)
Others	8 (5.9%)
The number of parathyroid gland	
<4	16 (11.9%)
≥ 4	119 (88.1%)
Blood pressure	
Pre-PTX systolic pressure (mmHg)	139.4 ± 22.8
Pre-PTX diastolic pressure (mmHg)	88.6 ± 15.4
1-year post-PTX systolic pressure (mmHg)	144.5 ± 23.5
1-year post-PTX diastolic pressure (mmHg)	80.1 ± 13.3
cardiovascular drugs used during the follow-up	
ACEI	5 (3.7%)
ARBs	30 (22.2%)
calcium blockers	50 (37.0%)
cardiotonics	1 (0.7%)
β-blockers	48 (35.6%)
isosorbide mononitrate	11 (8.1%)
trimetazidine	8 (5.9%)

*PD* peritoneal dialysis, *HD*: hemodialysis, *ESRD* End Stage Renal Disease, *CGN* chronic glomerulonephritis, *ACEI* angiotensin-converting enzyme inhibitors, *ARBs* angiotensin receptor blockers

The serum calcium and phosphate declined immediately after the PTX, and were maintained at a stable level after the PTX all along with the follow-up. The serum PTH declined immediately after the PTX and kept gradually increasing in the follow-up, after 1 year of the PTX, the PTH was 69.15 (27.95, 180.28) ng/L compared with a pre-operative level of 2357.00 (1709.00, 2777.00) ng/L. The serum alkaline phosphatase increased after the PTX, reached peak level after 1 month of the PTX, and gradually declined to normal level after 1 year of the PTX. The serum albumin declined after the PTX, and increased after 3 months of the PTX significantly (Table 2).

**Table 2:** Biochemical parameters change of ESRD patients pre- and post PTX

	pre-PTX	1 week Post-PTX	1 moth Post-PTX	3 moths Post-PTX	1 year Post-PTX
Ca (mmol/l),x ± s	2.56 ± 0.21	2.09 ± 0.27^a^	2.11 ± 0.28^a^	2.12 ± 0.31^a^	2.16 ± 0.26^a^
P (mmol/l),x ± s	2.15 ± 0.47	1.10 ± 0.42^a^	0.89 ± 0.33^a^	1.05 ± 0.45^a^	1.30 ± 0.49^a^
PTH (ng/l), M(0.25,0.75)	2357.00 (1709.00,2777.00)	14.20 (5.20,27.00)^a^	40.30 (21.15,77.40)^a^	74.60 (31.15,165.75)^a^	69.15 (27.95,180.28)^a^
ALP(u/l), M(0.25,0.75)	383.00 (188.00,792.00)	415.00 (235.50,1044.50)^a^	433.00 (197.50,634.00)^a^	136.50 (90.00,243.25)^a^	91.50 (66.75,139.25)^a^
ALB(g/l),x ± s	40.36 ± 5.14	37.16 ± 4.28^a^	39.83 ± 4.05	42.07 ± 4.68^a^	42.10 ± 4.65^a^

PS: compared with pre-PTX, *p*^a^ <0.05

The differences of biochemical parameters between pre and post-PTX were analyzed using paired t test and Wilcoxon signed-rank sum test

*Ca* serum calcium, *P* serum phosphate, *PTH* parathyroid hormone, *ALP* alkaline phosphatase, *ALB* albumin

### Change of ultrasonic cardiogram pre and post- PTX

In all the patients, the main ultrasonic cardiogram parameter dramatically improved compared with that at pre-PTX. The LVM and LVMI significantly decreased after 1 year of the PTX. The LVM was 172.82 (135.80, 212.91) g after 1 year of PTX, while LVM was 192.76 (157.56, 237.97) g pre- PTX (*p<*0.001). Furthermore, LVMI pre- PTX was 123.54 (105.49, 146.64) g/m^2^, after 1 year of PTX, the LVMI was 107.01 (86.79,128.42) g/m^2^ (*p<*0.001). The LVDd, PWd, LVDs, and IVSs also declined significantly after PTX (*P<*0.05). There was no statistical difference observed in EF%, FS%, LA, AO, IVSd, LVPWs as compared with those during pre-PTX (Table 3).

**Table 3:** Ultrasonic cardiogram parameter change of ESRD patients pre- and post- PTX (*n* = 135)

	pre-PTX	1 year Post-PTX	*P* value
LVM(g), M(0.25,0.75)	192.76 (157.56,237.97)	172.82 (135.80,212.91)	*p<*0.001
LVMI(g/m^2^),M(0.25,0.75)	123.54 (105.49,146.64)	107.01 (86.79,128.42)	*p<*0.001
EF(%),x ± s	65.52 ± 7.80	66.69 ± 7.07	*p* = 0.121
FS(%),x ± s	36.63 ± 5.80	37.01 ± 5.70	*p* = 0.420
LVDd (mm),x ± s	50.01 ± 6.26	48.69 ± 5.94	*p* = 0.018
IVST (mm), M(0.25,0.75)	10.20 (9.00, 11.95)	10.00 (9.00,11.00)	*p* = 0.075
PWd (mm), M(0.25,0.75)	10.90 (9.05,12.40)	10.00 (9.00,11.00)	*p =* 0.01
LA (mm),x ± s	34.55 ± 6.79	34.64 ± 6.02	*p* = 0.313
AO (mm),x ± s	30.83 ± 4.84	29.75 ± 4.23	*p* = 0.074
LVDs (mm),x ± s	31.74 ± 5.54	30.78 ± 5.32	*p* = 0.028
LVPWs (mm),x ± s	15.60 ± 2.52	14.91 ± 3.01	*p* = 0.237
IVSs (mm),x ± s	15.98 ± 3.22	14.33 ± 2.92	*p* = 0.001
dry weight (kg),x ± s	56.81 ± 10.95	57.72 ± 11.62	*p =* 0.01

The differences of ultrasonic cardiogram parameters between pre and post-PTX were analyzed using paired t test and Wilcoxon signed-rank sum test

*LVM* left ventricular mass, *LVMI* left ventricular mass index, *EF%* ejection fraction%, *FS%* fractional shortening%, *LVDd* left ventricular end-diastolic diameter, *IVST* interventricular septum thickness, *LVPWD* left ventricular posterior wall end-diastolic thickness, *LA* left atrial diameter, *AO* aorta, *LVDs* left ventricular end-systolic dimension, *LVPWs* left ventricular posterior wall end-systolic thickness, *IVSs* ventricular septal end-systolic thickness

Even though there was no statistical difference observed in EF%, FS%, the rising tendency could be observed. In the subgroup analysis of 35 patients with EF% ≤ 60% pre-PTX, EF%, and FS% significantly improved after 1 year of the PTX compared with pre-PTX (EF%, 64.90 ± 7.90% vs. 55.71 ± 4.78%, *p<*0.001; FS%, 35.48 ± 6.34% vs. 29.54 ± 2.88%, *p<*0.001) (Table 4).

**Table 4:** Ultrasonic cardiogram parameter change of ESRD patients whose EF% ≤ 60% pre- PTX (*n* = 35)

	pre-PTX	1 year Post-PTX	*P* value
EF(%),x ± s	55.71 ± 4.78	64.90 ± 7.90	*p<*0.001
FS(%),x ± s	29.54 ± 2.88	35.48 ± 6.34	*p<*0.001
LVM(g),M(0.25,0.75)	207.45 (176.18,278.46)	193.96 (148,87,251.60)	*p* = 0.035
LVMI(g/m^2^),M(0.25,0.75)	135.75 (110.13,178.11)	117.24 (102.15,159.40)	*p* = 0.013
LVDd (mm),x ± s	52.61 ± 6.87	50.79 ± 6.92	*p* = 0.117
IVST (mm),M(0.25,0.75)	11.15 (9.30,12.0)	10.00 (9.40,12.60)	*p* = 0.918
PWd (mm),M(0.25,0.75)	11.00 (9.85,12.93)	10.00 (9.00,11.30)	*p* = 0.245
LA (mm),x ± s	36.61 ± 8.66	36.70 ± 7.06	*p* = 0.592
AO (mm),x ± s	30.91 ± 5.14	29.89 ± 4.76	*p* = 0.423
LVDs (mm),x ± s	36.99 ± 6.10	33.34 ± 6.48	*p =* 0.001
LVPWs (mm),x ± s	15.59 ± 2.62	15.84 ± 3.01	*p* = 0.202
IVSs (mm),x ± s	16.19 ± 3.48	15.42 ± 2.97	*p* = 0.486
dry weight (kg),x ± s	56.28 ± 11.96	57.44 ± 11.96	*p =* 0.018

The differences of ultrasonic cardiogram parameters change of ESRD patients whose EF% ≤ 60% pre-PTX were analyzed using paired t test and Wilcoxon signed-rank sum test

*LVM* left ventricular mass, *LVMI* left ventricular mass index, *EF%* ejection fraction%, *FS%* fractional shortening%, *LVDd* left ventricular end-diastolic diameter, *IVST* interventricular septum thickness, *LVPWD* left ventricular posterior wall end-diastolic thickness, *LA* left atrial diameter, *AO* aorta, *LVDs* left ventricular end-systolic dimension, *LVPWs* left ventricular posterior wall end-systolic thickness, *IVSs* ventricular septal end-systolic thickness

### The influence factor for the overturn of LVH and the improvement of EF%

According to the diagnostic criteria of left ventricular hypertrophy (LVH), before the PTX, the incidence of LVH was 59.26% (80), and among them, 43.75% (35) patients recovered from LVH after 1 year of the PTX. To determine the reason for the overturn of LVH, we collected some data maybe related to the overturn of LVH including the cardiovascular drugs (ACEI, ARBs, calcium blockers, cardiotonics, β-blockers, isosorbide mononitrate, trimetazidine) used during the follow-up, and the blood pressure 1-year post-PTX, then we conducted the multiple logistic regression analyses. In the multiple logistic regression analyses, we didn’t observe the significant influence factor for the overturn of LVH (Table 5).

**Table 5:** The influence factor for the overturn of LVH in ESRD patients post-PTX on multiple logistic regression analysis (*n* = 80)

	OR(95%CI)	***P*** value
**Post-PTX**		
ACEI	0.749 (0.054, 10.452)	0.830
ARBs	1.672 (0.500, 5.592)	0.404
calcium blockers	1.868 (0.640, 5.448)	0.253
cardiotonics	–	–
β-blockers	2.500 (0.910, 6.872)	0.076
isosorbide mononitrate	1.136 (0.168, 7.657)	0.896
trimetazidine	0.512 (0.075, 3.487)	0.494
systolic pressure mmHg	1.010 (0.981,1.039)	0.519
diastolic pressure mmHg	1.003 (0.956, 1.053)	0.899

The influence factors for the overturn of LVH in ESRD patients 1-year post-PTX were analyzed using multiple logistic regression analysis, unadjusted

*ACEI* angiotensin-converting enzyme inhibitors, *ARBs* angiotensin receptor blockers

In patients whose EF% ≤ 60% (35), there were 82.86% (29) patients underwent an increase of 10% or greater in its EF% absolute value after 1 year of the PTX, which showed the markedly improvement of the LV systolic function. To determine why the EF% increased, we collected some data maybe related to the improvement of EF% including the cardiovascular drugs (ACEI, ARBs, calcium blockers, cardiotonics, β-blockers, isosorbide mononitrate, trimetazidine) used during the follow-up, and the blood pressure 1-year post-PTX, and then we conducted the multiple logistic regression analyses. In the multiple logistic regression analyses, we didn’t observe the significant relationship between cardiovascular drugs, blood pressure and the improvement of EF% either (Table 6).

**Table 6:** The influence factor for the improvement of the left ventricular systolic function in ESRD patients post-PTX on multiple logistic regression analysis (*n =* 35)

	OR(95%CI)	***P*** value
**Post-PTX**		
ACEI	–	–
ARBs	0.397 (0.027, 5.898)	0.502
calcium blockers	0.506 (0.020, 12.694)	0.679
cardiotonics	–	–
β-blockers	10.505 (0.728, 151.527)	0.084
isosorbide mononitrate	0.062 (0.000, 7.811)	0.259
trimetazidine	0.781 (0.064, 4.813)	0.996
systolic pressure mmHg	0.958 (0.887,1.034)	0.272
diastolic pressure mmHg	1.125 (0.965, 1.312)	0.133

The influence factors for the improvement of the left ventricular systolic function in ESRD patients 1-year post-PTX were analyed using multiple logistic regression analysis, unadjusted

*ACEI* angiotensin-converting enzyme inhibitors, *ARBs* angiotensin receptor blockers

## Discussion

SHPT, a common complication of ESRD, is a clinical condition associated with bone and mineral disorder, anemia, pruritus, hypertension, vascular calcification, **cardiovascular disease**, and sexual dysfunction [8]. **The morbidity and mortality of cardiovascular disease in dialysis patients is found significantly high,** and the CVD accounts for about 50% of death in hemodialysis patients in European countries and the USA [9–11]. Over the past decades, evidence has indicated that ESRD might induce myocardial ischemia and left ventricular dysfunction [12]. Some findings have consistently shown that progressive LVH and cardiac fibrosis may cause diastolic and systolic dysfunction, but the pathophysiologic mechanism is still poorly understood [13]. Several previous studies have documented that successful PTX is associated with reduced all-cause mortality and cardiovascular mortality [3, 14, 15].

LVH and left ventricular diastolic dysfunction are the most common types of myocyte damage noticed in uremic patients. LVH is an adaptive response to pressure and volume overload. In dialysis patients, the incidence of LVH was reported about 68–89%, and it has been reported that LVMI increases substantially in the majority of dialysis patients who are treated with standard care, including angiotensin blockade, dialysis frequency, and anemia management [16], and LVMI was associated with a higher CVD mortality [1]. PTH is regarded as a uremic toxin as it could aggravate renal anemia and damage myocyte in hemodialysis patients. Previous studies have shown that the serum PTH level is independently associated with the LVH [17], with the decline of PTH, the anemia alleviated, and the nutrition condition improved, and the toxicity of PTH receded. Recent experimental and clinical studies have highlighted the crucial role of fibroblast growth factor 23 (FGF23) receptor, Vitamin D deficiency, systolic blood pressure in induction and progression of LVH in CKD patients [17–19]. A study showed the PTX could improve blood pressure by better control of BP, decrease afterload, and improve systolic function [20].

Of all the patients, 43.75% diagnosed with LVH pre-PTX recovered from LVH after 1 year of PTX in our study, and we didn’t observe the significant influence factor for the overturn of LVH post-PTX including cardiovascular drugs and blood pressure in the multiple logistic regression analysis, so it can be confirmed the impact of PTX on the overturn of LVH further.

15% incidence of systolic dysfunction among newly started dialysis patients was reported, and there are case reports documenting an increase in EF% post-PTX [4, 21]. Small sample research conducted by N. Goto indicated that ten patients’ EF% had markedly improved after 6 months of PTX [5]. Traditional perspective studies confirmed that PTH is a promotion factor of LVH, while A. J. van Ballegooijen emphasized that the high concentration of PTH was significantly associated with low EF% [22]. Lowering PTH levels can ameliorate the arterial calcification, decrease the afterload, and improve the EF% [22].

In our study, we observed a generally rising trend in EF% and FS%, though there was no statistical difference observed in all patients. EF% was 66.69 ± 7.07% vs. 65.52 ± 7.80% and FS% was 37.01 ± 5.70% vs. 36.63 ± 5.80%. In the subgroup analysis of EF% ≤ 60% pre-PTX, we found that EF% and FS% after PTX significantly improved compared with pre-PTX and EF% level of 64.90 ± 7.90% vs. 55.71 ± 4.78%. And in these patients, 82.86% patients underwent an increase of 10% or greater in its EF% absolute value post 1 year of PTX, showing the markedly improvement of the LV systolic function. And in the multiple logistic regression analysis, we didn’t observe the significant relationship between the cardiovascular drugs, the blood pressure and the improvement of EF%, so we can affirm the effect of PTX on the improvement of the left ventricular systolic function. Compared with N Goto’s study, we conducted a longer and more comprehensive study.

By comparing our findings with previous ones, we conclude that the left ventricular function improved in ESRD patient after the PTX might be related to the causes, such as the decline of PTH after the PTX reduced or even overturned the damage of myocardium and ameliorated arterial calcification; the improvement of serum calcium and phosphorus abnormal also alleviated the vascular calcification; the improvement of nutrition status after the PTX was conducive to the recovery of the myocardium; PTX could improve blood pressure, with the better control of blood volume, the afterload declined, but in our study, the effect of BP didn’t observe in multiple logistic regression analysis.

## Conclusions

tPTX+AT is an effective curative intervention of secondary hyperparathyroidism and can significantly overturn the LVH and increase the left ventricular systolic function.

### Limitations

Our study has some limitations: this is a single-center retrospective study, and we could not set randomize control group, which makes the study rigorous enough; the sample capacity was not large, especially the subgroup; the confounding bias interference factors were not excluded thoroughly.

## Data Availability

The datasets created during and/or analyzed during the current study available from the corresponding author on reasonable request.

## Abbreviations

SHPT: Secondary hyperparathyroidism
ESRD: End-stage renal disease
PTX: Parathyroidectomy
LVH: Left ventricular hypertrophy
LVM: Left ventricular mass
LVMI: Left ventricular mass index
tPTX+AT: Total PTX with forearm autotransplantation
CVD: Cardiovascular disease
KDIGO: Kidney Disease Improving Global Outcomes
PTH: Parathyroid hormone
ACEI: Angiotensin-converting enzyme inhibitors
ARBs: Angiotensin-receptor blockers
EF%: Ejection fraction%
FS%: Fractional shortening%
LVDd: Left ventricular end-diastolic diameter
IVST: Interventricular septum thickness
LVPWD: Left ventricular posterior wall end-diastolic thickness
LA: Left atrial diameter
AO: Aorta
LVDs: Left ventricular end-systolic dimension
LVPWs: Left ventricular posterior wall end-systolic thickness
IVSs: Ventricular septal end-systolic thickness
BSA: Body surface area
SPSS: Statistical package for the social sciences
KS test: Kolmogorov-Smirnov test
the USA: The United States of America
FGF23: Fibroblast growth factor 23
